# Effectiveness of CoronaVac in the prevention of COVID-19, a test-negative case-control study in Brazil

**DOI:** 10.1016/j.bjid.2024.103856

**Published:** 2024-08-05

**Authors:** Expedito J.A. Luna, José C. Moraes, Manuela A. Roediger, Erique J.F.P. Miranda, Patrícia E. Braga, João I.D. França, Pedro H.M. Pacheco, Marcos Alves de Lima, Lucas Ragiotto, Eliana N.C. Barros, the CoronaVac Effectiveness Study Group

**Affiliations:** a Universidade de São Paulo, Faculdade de Medicina, São Paulo, SP, Brazil; b Faculdade de Ciências Médicas da Santa Casa, São Paulo, SP, Brazil; c Instituto Butantan, São Paulo, SP, Brazil

**Keywords:** SARS-CoV2, Inactivated vaccines, Effectiveness, Test-negative, Case-control study, Brazil

## Abstract

The present study aimed to evaluate the effectiveness of two doses of CoronaVac in preventing SARS-CoV-2 symptomatic disease with virological confirmation, as well as in the prevention of COVID-19 moderate and severe cases. A test-negative unmatched case-control design was used, in which cases were patients with suspected COVID-19 (presenting at least two of the following symptoms: fever, chills, sore throat, headache, cough, runny nose, olfactory or taste disorders) with virological confirmation, and controls were those whose SARS-CoV-2 test was negative. As for exposure, participants were classified as unvaccinated, or vaccinated with a complete schedule. Suspected COVID-19 cases were identified from March to November 2021, in two cities located in the State of São Paulo, Brazil. All participants signed the Informed Consent Form before enrollment. RT-PCR results and vaccination data were obtained from the local surveillance systems. Up to two phone calls were made to obtain information on the outcome of the cases. A total of 2981 potential participants were screened for eligibility, of which 2163 were included, being 493 cases and 1670 controls. Vaccination, age, the reported contact with a COVID-19 suspected or confirmed case in the 14 days before symptoms onset, and the educational level were the variables independently associated with the outcome. The adjusted vaccine effectiveness for symptomatic COVID-19 (AVE) was 39.0 % (95 % CI 6.0–60.0 %). The AVE in the prevention of moderate and severe disease was 91.0 % (95 % CI 76.0–97.0 %). Our results were influenced by the waning of the Gamma variant, in the second trimester of 2021, followed by the increase in vaccination coverage, and a drop in the number of cases in the second half of the year. The study demonstrated the high effectiveness of CoronaVac in preventing moderate/severe COVID-19 cases.

## Background

The incorporation of vaccines into the arsenal of control measures, at the end of 2020, changed the course of the COVID-19 pandemic. A reduction in the incidence of the disease was observed, particularly in severe cases, hospitalizations and deaths.

An unprecedented effort in history by the academic community, research funding agencies, health care services, governments and the pharmaceutical industry, enabled the short-term development of safe and effective vaccines against SARS-CoV-2. The consultation of the WHO SARS-CoV-2 vaccine landscape demonstrates the strength of this effort. Almost 400 vaccine candidates were in different stages of development as of March 2023, of which 180 had reached the clinical investigation phase, and more than thirty were already on the market.^1^ Several platforms have been used in their development and manufacturing, some new, such as mRNA vaccines, viral vector vaccines, and protein subunit vaccines, as well as other technologies already widely used in vaccines’ manufacturing for other infectious agents, such as inactivated vaccines.

CoronaVac, an adjuvanted inactivated virus vaccine, developed by the pharmaceutical company SINOVAC, in partnership with the Butantan Institute, was the first vaccine available in Brazil. Phase 3 trials demonstrated its safety, and an efficacy in preventing symptomatic disease caused by SARS-CoV-2 from 50.7 % (95 % CI 36.0–62.0 %) in Brazil,^2^ to 83.5 % (95 % CI 65.4–92.1 %) in Turkey.^3^ Safety and efficacy data are fundamental for the analysis of a product by regulatory agencies, which decide on authorization for the commercialization and use of the vaccine, but it is data on the effectiveness of the vaccine that allow the evaluation of the product's performance in real life.

The vaccination campaign against COVID-19 in Brazil began in January 2021. As of the second half of the year, studies on the effectiveness of the vaccination program began to be published. The vast majority of them used data routinely collected by national health information systems, specifically the mandatory reporting diseases system and the vaccination registry. Although they are able to provide relevant information, effectiveness studies with secondary data are limited in terms of the completeness and quality of the data, and usually demand probabilistic linking of database records. In order to overcome these limitations, the present study aimed to evaluate the effectiveness of two doses of CoronaVac in preventing symptomatic disease with virological confirmation by SARS-CoV-2, as well as COVID-19 moderate and severe cases.

## Methods

A test-negative unmatched case-control design was used, in which cases were patients with suspected COVID-19 with virological confirmation, and controls were those whose test was negative for SARS-CoV-2.

Cases and controls were obtained at primary health care and emergency care clinics or from COVID-19 surveillance database with the onset symptoms registered from March to November 2021 from two municipalities located in the state of São Paulo, Brazil: Campinas (population 1,223,237 in 2021) and Araraquara (population 240,542 in 2021).^6^

The eligibility criteria were: being a clinically suspected COVID-19 case, according to the Brazilian Ministry of Health definition (presenting at least two of the following symptoms: fever, chills, sore throat, headache, cough, runny nose, olfactory or taste disorders)^4^; having performed the RT-PCR test with a sample collected within 7 days of the onset of symptoms; being 18 years or older, and having had the chance to receive two doses of the CoronaVac vaccine in the basic scheme, in accordance with the vaccination strategy of the municipality of residence. The exclusion criteria were to have a contraindication to the use of any vaccine against COVID-19; and having received any COVID-19 vaccine other than CoronaVac in the basic scheme.

### Definition of cases and controls

Case: Suspected COVID-19 case confirmed by laboratory criteria (positive RT-PCR test result).

Control: Suspected COVID-19 case discarded by laboratory criteria (negative RT-PCR test result).

Clinical severity classification: After their enrollment, participants were classified according to WHO's COVID-19 clinical severity scale.^5^ Participants with score above 3 were classified as moderate/severe.

### Definition of vaccine status

Complete schedule: Having received two doses of CoronaVac in the basic schedule with an interval of at least 14 days between the second dose and the onset of symptoms suggestive of COVID-19.

Unvaccinated: Not having received any dose of COVID-19 vaccine or having received only one dose with an interval of less than 14 days between the first dose and the onset of symptoms suggestive of COVID-19.

### Ethical issues

The study was approved by the Santa Casa de São Paulo ethical review board (statement number: 4.734.648). The participant's informed consent was obtained prior to performing any study procedure. The potential participants or legal guardians were only included in the study after their agreement to participate and signing the Informed Consent Form (ICF). Participants identified in the surveillance databases (retrospective approach) were invited to participate in the study through telephone calls. The calls were recorded, and the ICF was read and explained. The participant's consent was verbally obtained. The study obtained a waiver of the ICF from the IRB to include deceased cases.

### Procedures

A screening questionnaire was applied to those individuals who attended the healthcare clinics with a clinical suspicion of COVID-19. After validation of the inclusion and exclusion criteria, eligible participants were invited to participate in the study. Upon acceptance, they proceeded to a face-to-face questionnaire and up to two telephone interviews to update the clinical evolution of the disease, or consultation of their medical records. For participants identified in the databases, a single questionnaire was used, applied by a telephone interview.

### Data management and analysis

Data from the participants’ interviews were recorded in electronic questionnaires, on the REDCap platform (REDCap Consortium, Nashville, TN, USA). Two national health information systems, managed by the Brazilian Ministry of Health and by the State of São Paulo's Health Department, were used to obtain additional data and/or to validate the participants’ responses: the Gerenciamento de Atividades Laboratoriais – GAL (Management of Laboratory Activities, a public health laboratory database), to get the SARS-CoV-2 PCR results, and the VaciVida (State of São Paulo's vaccine registry), to access the COVID-19 vaccine data (vaccine manufacturer and vaccination dates).

Additionally, the participants medical records were consulted, if necessary, to obtain data on the evolution and outcomes of the disease. The data were aggregated into a study database and analyzed using Stata 16.0 (StataCorp LP, College Station, TX, USA), R 4.2 (R Foundation, Vienna, Austria) and SPSS 25.0 (IBM Corp. Released 2017. Armonk, NY, USA).

The measure of effect was the Vaccine Effectiveness (VE). It was calculated by the formula: VE = 1 ‒ Odds Ratio, and presented with its 95 % Confidence Interval. In the effectiveness analysis for symptomatic virologically confirmed COVID-19 cases the dependent variable was the status, as a case or a control. The exposure variable was the vaccination status, and the co-variables were: age, sex, race/ethnicity, educational level, Body Mass Index (BMI), comorbidities, and variables related to exposure to SARS-CoV-2. In the effectiveness for moderate/severe disease, just the cases were included. The dependent variable was a score greater than 3 in the case severity classification, compared to those with a score equal to 3 or lower.^5^ Participants with incomplete schedule were not included in the VE analysis.

The descriptive analysis was conducted. Differences in proportions between cases and controls were analyzed using the Chi-Square test or χ^2^ for linear trend. The *t*-test was used to determine differences between means. Logistic regression analysis was conducted to estimate the crude odds ratio of the dependent variable for each variable of interest. Variables with *p* < 0.10 or with clinical relevance were incorporated into the regression analysis. Logistic regression analysis for unmatched studies to control for confounding variables was conducted to obtain adjusted odds ratios and their 95 % Confidence Intervals. The variables with *p* < 0.05, the statistical adjustment variables and those that were deemed of clinical relevance were kept in the final model.

## Results

**Fig. 1:**
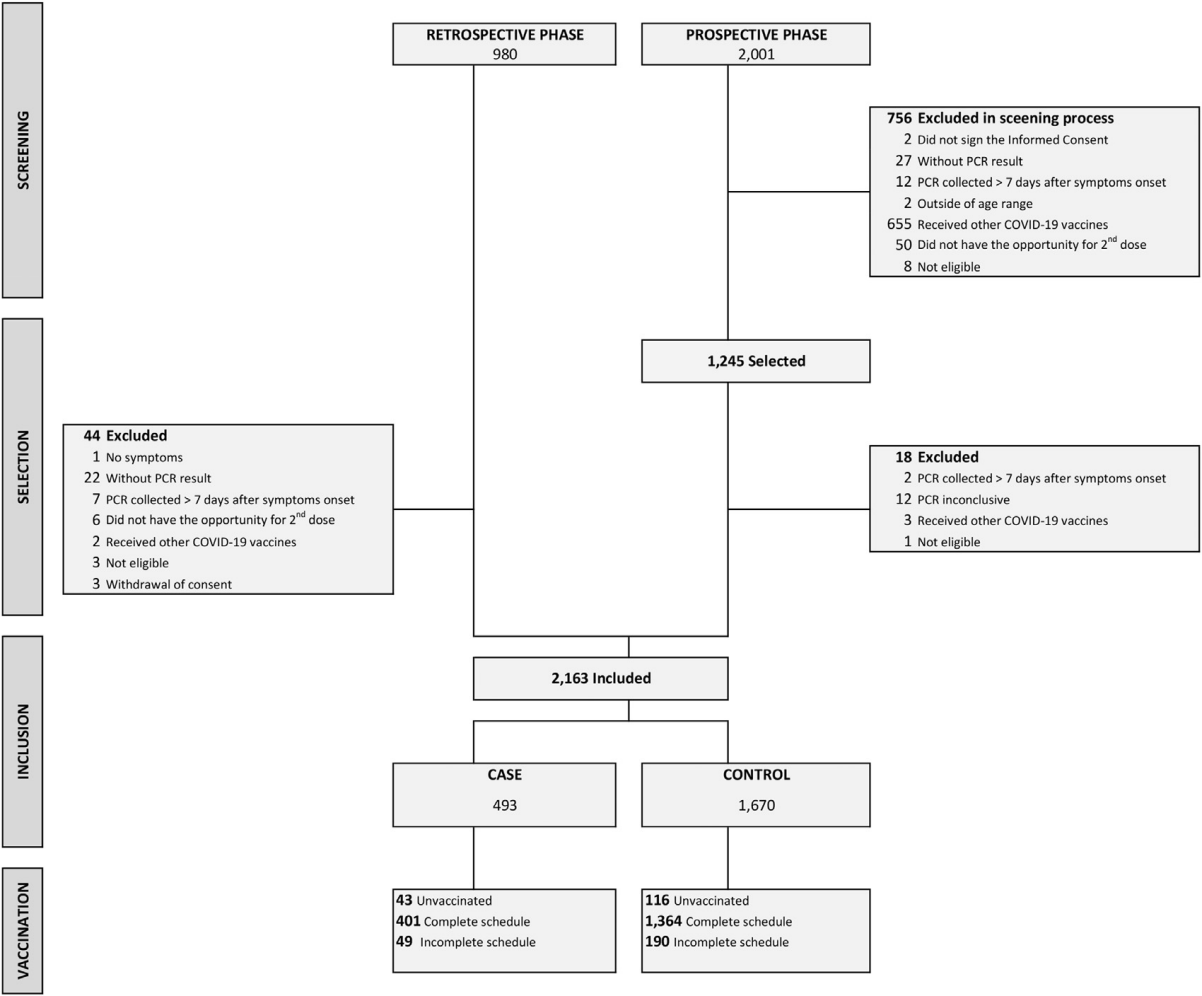
Participants flowchart.

A total of 2981 potential participants were screened for eligibility, of which 2163 were actually included, being 493 cases and 1670 controls (Fig. 1).

Cases were older and less educated than controls (Table 1). They also were more likely to have had contact with someone with suspected or confirmed COVID-19 in the two weeks before the onset of symptoms (Table 2). Regarding their vaccine status, cases and controls were classified as unvaccinated, vaccinated with a complete schedule, or vaccinated with an incomplete schedule (Table 2). For vaccinated participants, the median interval between the second dose and the onset of symptoms was 110 days (Results are not show).

**Table 1:** Characteristics of cases and controls.

Variables	Total (*n* = 2163)		Cases (*n* = 493)		Controls (*n* = 1670)		*p*-value
	n	%	n	%	n	%	
Age (years)							
Mean	43.85		52.33		41.34		<0.001^a^
Sex							
Female	1405	65.0	302	61.3	1103	66.0	0.053
Male	758	35.0	191	38.7	567	34.0	
Race/Ethnicity (*n* = 2081)							
White	1333	64.1	315	65.2	1018	63.7	0.552^b^
Black	233	11.2	51	10.6	182	11.4	
Mixed	503	24.2	114	23.6	389	24.3	
Asian	11	0.5	3	0.6	8	0.5	
Indigenous	1	0.0	0	0.0	1	0.1	
BMI (kg/m²) (*n* = 1968)							
Median (Q_1_‒Q_3_)	26.5	(23.6‒30.1)	27.0	(23.7‒30.1)	26.5	(23.5‒30.2)	0.398
BMI (kg/m^2^) (*n* = 1968)							
< 30	1454	73.9	302	74.2	1152	73.8	0.899
≥ 30	514	26.1	105	25.8	409	26.2	
Educational level (*n* = 2029)							
Illiterate	45	2.2	21	4.9	24	1.5	<0.001^c^
Incomplete primary education	175	8.6	65	15.3	110	6.9	
Complete primary education	123	6.1	30	7.0	93	5.8	
Incomplete high school	105	5.2	24	5.6	81	5.1	
Complete high school	643	31.7	122	28.6	521	32.5	
Technical education	145	7.1	24	5.6	121	7.5	
Incomplete undergraduate studies	205	10.1	25	5.9	180	11.2	
Complete undergraduate studies	449	22.1	82	19.2	367	22.9	
Graduate studies	139	6.9	33	7.7	106	6.6	
Municipality (*n* = 2163)							
Araraquara	1239	57.3	223	45.2	1016	60.8	<0.001
Campinas	924	42.7	270	54.8	654	39.2	

BMI, Body Mass Index, ratio of the individual's weight to the square of his/her height; Q_1_–Q_3_, First quartile; Third quartile.

a *t*-test.

b White *×* Non-white.

c (Illiterate + Primary) × (High + Technical + Undergraduate + Graduate).

**Table 2:** Distribution of cases and controls according to variables related to exposure to SARS-CoV-2 and vaccination with CoronaVac.

Variables	Total^a^		Cases^a^		Controls^a^		*p*-value^b^
	n	%	n	%	n	%	
Contact with COVID-19 suspected or confirmed cases in the 14 days before symptoms onset (*n* = 2120)							<0.001
No	1190	56.1	217	31.6	973	59.1	
Yes	930	43.9	257	68.4	673	40.9	
Uses public transportation (*n* = 1914)^c^							0.119
Never	1318	68.9	305	72.6	1013	67.8	
Occasionally	234	12.2	41	9.8	193	12.9	
Always	362	18.9	74	17.6	288	19.3	
Number of people in household (*n* = 1914)							0.605
0–3	1206	37.0	262	61.9	944	63.3	
4 and more	708	63.0	161	38.1	547	36.7	
Uses face mask (*n* = 1899)^c^							0.496
Always	1756	92.5	384	93.4	1372	92.2	
Sometimes	107	5.6	22	5.4	85	5.7	
Never	36	1.9	5	1.2	31	2.1	
Changes mask every 2 h (*n* = 1889)^c^							0.058
Always	605	32.0	146	35.8	459	31.0	
Sometimes	539	28.5	121	29.7	418	28.2	
Never	745	39.4	141	34.6	604	40.8	
Avoids crowded places (*n* = 1897)^c^							0.135
Always	1357	71.5	303	74.1	1054	70.8	
Sometimes	434	22.9	91	22.2	343	23.1	
Never	106	5.6	15	3.7	91	6.1	
Cleans hands with alcohol gel after contact with surfaces (*n* = 1894)^c^							0.097
Always	1497	79.0	338	82.6	1159	78.0	
Sometimes	274	14.5	52	12.7	222	14.9	
Never	123	6.5	19	4.6	104	7.0	
Vaccine schedule (*n* = 2163)							0.308
Unvaccinated	159	7.4	43	8.7	116	7.0	
Complete	1765	81.6	401	81.3	1364	81.6	
Incomplete	239	11.0	49	10.0	190	11.4	
Clinical severity (*n* = 2076)							0.001
Mild	1840	88.6	359	74.0	1481	93.1	
Moderate/Severe	236	11.4	126	26.0	110	6.9	

a Missing data not included in the analysis.

b Chi-Squared test.

c χ^2^ for linear trend.

Cases were more likely to refer chronic comorbidities than controls: diabetes mellitus, hypertension, heart disease and pulmonary disease (Table S1).

In the univariate analysis, the variables that were significantly associated with the odds of being a case were age, the educational level, any previous comorbidity, and the reported contact with a COVID-19 suspected or confirmed case in the 14 days before symptoms onset (Tables 1, 2 and S1). Regarding the clinical severity classification, the vast majority of controls were classified as mild. As for COVID-19 cases, almost a quarter of them were classified as moderate/severe (Table 2 and S2).

In the multivariate logistic regression analysis, the age, the reported contact with a COVID-19 suspected or confirmed case in the 14 days before symptoms onset, and the educational level remained in the model, being independently associated with the outcome (Table 3). Finally, the Adjusted Vaccine Effectiveness (AVE) for symptomatic virologically confirmed COVID-19 was 38.9 % (95 % CI 5.9 %‒60.4 %) (Table 4).

**Table 3:** Adjusted odds ratio for the association of independent variables and cases/controls.

Variables	OR (95 % CI)	*p*-value
Vaccine Schedule		
Complete	0.61 (0.40–0.94)	0.025
Unvaccinated	1.0 (ref)	–
Age (years)	1.02 (1.01–1.03)	<0.001
Education		
Illiterate+Primary	1.45 (1.06–2.00)	0.022
High school+Undergraduate+Graduate	1.0 (ref)	–
Contact with a COVID-19 suspected or confirmed case		
No	0.46 (0.37–0.58)	<0.001
Yes	1.0 (ref)	–
Comorbidity		
Yes	1.08 (0.81–1.43)	0.615
No	1.0 (ref)	–

**Table 4:** Adjusted vaccine effectiveness among participants who received CoronaVac in the complete or incomplete vaccination schedules in relation to those who were not vaccinated; and according to disease severity (moderate/severe cases × mild cases).

Variables	Adjusted vaccine effectiveness		
	All participants		
	VE	95 % CI	*p*-value
Complete schedule	38.9 %	5.9–60.4 %	0.025
	Moderate/severe cases × mild cases		
Complete schedule	91.4 %	75.6–96.9 %	< 0.001

The multivariate model was adjusted comparing moderate/severe cases to the mild ones. In this analysis, vaccination with complete schedule was independently associated with severity, along with age, educational level, contact with a suspected or confirmed case in the 14 days before symptoms onset, and reporting any comorbidity (Table 5). Adjusted vaccine effectiveness for moderate/severe cases was 91.4 % (95 % CI 75.6 %‒96.9 %) with a complete schedule (Table 4).

**Table 5:** Adjusted odds ratio for the association of independent variables and moderate/severe cases and mild cases × controls.

Variables	OR (95 % CI)	*p*-value
Vaccine Schedule		
Complete	0.09 (0.03–0.24)	<0.001
Unvaccinated	1.0 (ref)	–
Age (years)	1.12 (1.09–1.14)	<0.001
Education		
Illiterate+Primary	1.80 (1.02–3.18)	0.041
High school+Undergraduate +Graduate	1.0 (ref)	–
Contact with a COVID-19 suspected or confirmed case		
No	1.78 (1.03–3.06)	0.037
Yes	1.0 (ref)	–
Comorbidity		
Yes	2.97 (1.56–5.62)	<0.001
No	1.0 (ref)	–

## Discussion

In this test-negative case-control study, effectiveness of CoronaVac in the prevention of symptomatic virologically confirmed COVID-19 was 39 %. These figures are in line with the results of other CoronaVac effectiveness studies. In the State of São Paulo, Brazil, in early 2021, effectiveness of CoronaVac among older adults was estimated in 46.8 % (95 % CI 38.7–53.8 %).^7^ In Hong Kong, the effectiveness of CoronaVac in the prevention of mild to moderate disease among adults from 20 to 59 years of age was 25.1 % (95 % CI 14.7–34.3 %).^8^ In Guangdong, China, effectiveness against infection was 60.4 % (95 % CI 31.8–88.9 %) during an outbreak of the Delta variant.^9^ However, in Chile, the effectiveness of CoronaVac in the prevention of symptomatic virologically confirmed COVID-19 was higher (VE = 65.9 %; 95 % CI 65.2–66.6 %) in a study conducted in a short term after the vaccination campaign.^10^

Regarding moderate/severe disease, a high effectiveness was observed. Our result is consistent with the results of other studies. In a large study carried out with secondary data in Brazil, the effectiveness of CoronaVac for hospitalization due to COVID-19 was 95.6 % (95 % CI 82.4–99.9 %).^11^ Among older adults in the State of São Paulo, Brazil, effectiveness was 77.6 % against hospitalization, and 83.9 % against death.^7^ In Chile, effectiveness was 87.5 %, 90.3 % and 86.3 % against hospitalization, ICU admission, and death, respectively [10] while in Hong Kong, effectiveness of Coronavac in the prevention of severe disease and death was 69.9 %.^8^

Effectiveness estimates are usually lower than efficacy figures obtained in clinical trials. Efficacy of CoronaVac was 50.7 % in a study among frontline healthcare workers in Brazil (Palácios et al.); 65.3 % and 83.5 % among healthy adults from 18 to 59 years of age in Indonesia and Turkey, respectively.^3^^,^^12^ It has been documented that SARS-CoV-2 vaccines protection wanes over time.^13^ A study carried out in China showed that the effectiveness of CoronaVac against symptomatic infection decreased after six months, but remained high for serious outcomes, such as death.^14^

When interpreting and comparing different effectiveness studies, it is important to consider some aspects that may influence their results. The design of the study, the analyzed outcomes, the population or population subgroup included the period when the study was carried out, in relation to the evolution of the epidemic, as well as the follow-up time of the participants, should be taken into account. The present study was conducted right after the rollout of SARS-CoV-2 vaccines in Brazil, in early 2021. During the first trimester of 2021 Brazil experienced a large increase in the number of new COVID-19 cases and deaths, corresponding to the emergence of the Gamma variant.^15^ With time and the increase in vaccination coverage, the Gamma variant waned, and there was a drop in the number of cases in the second half of the year, when the field work of our prospective phase begun, and the predominant variant shifted to Delta. Older adults were the among the priority groups for vaccination, being the first ones to be vaccinated. This may have reduced the incidence and severity of cases among the population over 60 years of age, which accounted for just 20 % of study participants (data not shown). Regarding the follow-up time, among the participants for whom this variable can be computed, it was between 15 and 16 weeks. The study design in the prospective phase allowed the inclusion of a large proportion of eligible patients, the number of refusals was minimal.

In addition to the limitations arising from the observational design, our study has some others that deserve to be mentioned. Our sample size was small, not allowing us to estimate effectiveness by age group, especially for the older population. The number of severe cases was also small, and there was a higher concentration of deaths among subjects identified from the surveillance database, as the IRB exempted us from obtaining the informed consent form for their inclusion in the study.

In summary, the study demonstrated the high effectiveness of CoronaVac in preventing moderate and severe cases of COVID-19. Its effectiveness against virologically confirmed symptomatic infection was within expectations, similar to that observed in other studies, in the pre-Omicron period. The inactivated vaccine platform is very well known, representing lower costs than other COVID-19 vaccine's platforms, and has a high potential and capability for the national manufacturing to provide sustainable supply to the Ministry of Health.

## Conflicts of interest

The authors declare no conflicts of interest.

## Appendix Supplementary materials

Image, application 1

## Footnotes

Supplementary material associated with this article can be found in the online version at doi:10.1016/j.bjid.2024.103856.
