# Supplementary material for: Effectiveness of CoronaVac in the prevention of COVID-19, a test-negative case-control study in Brazil
Source: Braz J Infect Dis. 2024 Aug 5;28(5):103856. doi: 10.1016/j.bjid.2024.103856 (PMC11363480; doi:10.1016/j.bjid.2024.103856)
Supplement: Supplementary file 1 [file mmc1.docx]

BJID-D-24-00044_**Supplementary material**

**Supplementary material**

**Table S1** Distribution of cases and controls according to pre-existing comorbidities.

| **Comorbidity** | **Total** |  | **Cases** |  | **Controls** |  | **p** |
| --- | --- | --- | --- | --- | --- | --- | --- |
|  | **n** | **%** | **n** | **%** | **n** | **%** |  |
| Diabetes mellitus | 231 | 10.7 | 88 | 17.9 | 143 | 8.6 | **<0.001** |
| Hypertension | 443 | 20.5 | 141 | 28.7 | 302 | 18.1 | **<0.001** |
| Heart disease | 147 | 6.8 | 69 | 14.0 | 78 | 4.7 | **<0.001** |
| Pulmonary disease | 147 | 6.8 | 45 | 9.2 | 102 | 6.1 | **0.024** |
| Chronic kidney disease | 50 | 2.3 | 17 | 3.5 | 33 | 2.0 | 0.061 |
| Neurological disease | 79 | 3.7 | 25 | 5.1 | 54 | 3.3 | 0.074 |
| Primary immunodeficiency | 13 | 0.6 | 3 | 0.6 | 10 | 0.6 | >0.999 |
| HIV | 6 | 0.3 | 1 | 0.2 | 5 | 0.3 | >0.999 |
| Cancer | 24 | 1.1 | 8 | 1.6 | 16 | 1.0 | 0.222 |
| Transplantation | 2 | 0.1 | 0 | 0.0 | 2 | 0.1 | >0.999 |
| Any comorbidity | 643^a^ | 29.8 | 208 | 42.3 | 435 | 26.1 | <0.001 |

^a^ Some participants reported more than one comorbidity.

**Table S2** Distribution of cases and controls according to clinical severity score.

| **Score** | **Controls, n (%)** | **Cases, n (%)** | **Total, n (%)** |
| --- | --- | --- | --- |
| 3 | 1,481 (93.1) | 359 (74.0) | 1,840 (88.6) |
| 4 | 6 (0.4) | 6 (1.2) | 12 (0.6) |
| 5 | 7 (0.4) | 24 (4.9) | 31 (1.5) |
| 6 | ­ – | 3 (0.6) | 3 (0.1) |
| 7/8 | 1 (0.1) | 1 (0.2) | 2 (0.1) |
| 10 | 96 (6.0) | 92 (19.0) | 188 (9.1) |
| Total | 1,591 (100.0) | 485 (100.0) | 2,076 (100.0) |
